# Spatially Resolved Proteomic Mapping in Skin Organoid for Hair Follicle Development

**DOI:** 10.1016/j.mcpro.2025.101482

**Published:** 2025-12-09

**Authors:** Luling Liang, Jia Zhou, Wenjuan Wang, Wenwen Wang, Yi Liu, Jun Li, Ling Leng

**Affiliations:** 1 Institute of Clinical Medicine, State Key Laboratory of Complex, Severe, and Rare Diseases, Peking Union Medical College Hospital, Beijing, China; 2 Department of Dermatology, The First Medical Centre, Chinese PLA General Hospital, Beijing, China; 3 Department of Dermatology, State Key Laboratory of Complex, Severe, and Rare Diseases, Peking Union Medical College Hospital, National Clinical Research Center for Dermatologic and Immunologic Disease, Beijing, China

**Keywords:** hair follicle, skin organoid, spatially resolved proteome, development

## Abstract

Hair follicle development is a complex, highly regulated process involving interactions between epithelial and mesenchymal cells. However, the specific molecular mechanisms and important biological processes of hair follicle development remain poorly understood. How the extracellular matrix is involved in the hair follicle formation from hair germs remains to be investigated. In this study, we applied spatially resolved proteomic mapping to investigate the process of hair follicle development in skin organoids at different stages: D55, D75, D90, D140, D150, and D170, which corresponds to that from hair germ formation to hair follicle aging. Our analysis identified dynamic changes in protein expression and active protein synthesis during hair follicle appearance. We observed stage-specific protein expression patterns, with hair germ and hair peg formation, enriched in proteins involved in RNA processing and lipid metabolism. Meanwhile, hair follicle initial and full maturation highlighted proteins related to keratinization and extracellular matrix organization. Notably, trend proteins involved in keratinization and neuron–neuron synaptic transmission were upregulated from hair germ formation to the hair follicle appearance. We also found that CSNK1A1 and SFN exhibit abnormal expression in the hair follicles of patients with cicatricial alopecia, which further proves the role of CSNK1A1 and SFN in the normal development of hair follicles. The results provide a comprehensive spatial proteomic map of hair follicle development and offer new insights into the biological process driving hair follicle formation and maturation. These findings may guide future therapeutic strategies for hair regeneration and the treatment of hair disorders.

## Highlights

• Spatiotemporal proteome map of hair follicle development in skin organoids.
• Identification of stage-specific proteins and dynamic proteomic changes.
• Clarification of key molecular processes involved in hair follicle development.

## Graphical Abstract

**Figure:**
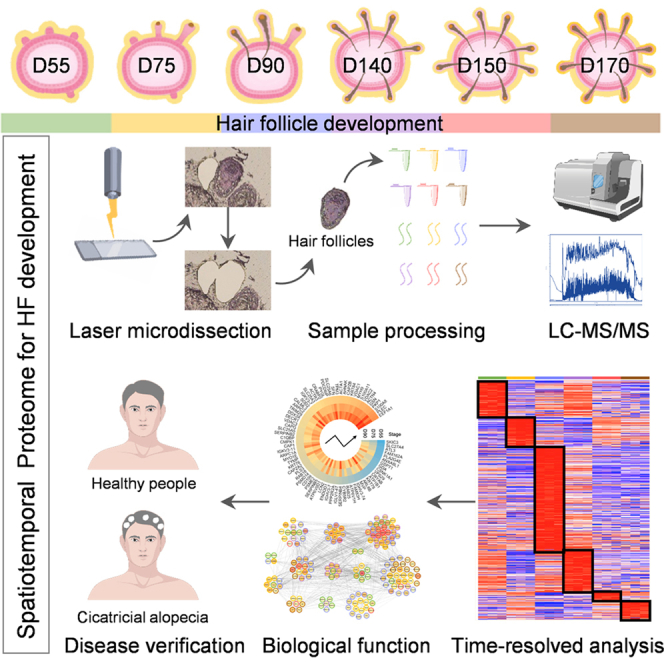

## In Brief

We applied spatially resolved proteomic mapping to study hair follicle development in skin organoids at different stages: hair germ formation, hair peg formation, hair follicle appearance, hair follicle initial maturation, hair follicle full maturation, and hair follicle aging. Stage-specific proteins and dynamic changes of proteins in a time series of hair follicle development were identified to clarify key molecular mechanisms involved in hair follicle formation and maturation.

Hair follicles are complex miniorgans produced by the interaction of epithelial and mesenchymal cells during embryonic development and the postnatal growth cycle. These structures are responsible for producing hair in cycles (1). Hair follicles are located in the papillary layer of the dermis and consist of the hair shaft, inner root sheath (IRS), outer root sheath, and connective tissue root sheath from the inside out. The base of the hair follicle is the bulb, which contains the dermal papilla, hair matrix cells, and melanocytes (2). The anagen of hair follicle development is a highly regulated process that contains three steps: induction, organogenesis, and cytodifferentiation, and these three steps include eight stages. The induction process is characterized by the interaction between epithelial and mesenchymal cells, containing the first stage, which is the formation of the placode by epithelial cell invagination. The organogenesis of the hair follicle consists of stages 2 to 5. The placode depresses into the dermis to form a hair germ first, which subsequently sinks deeper to form a solid columnar structure. The cytodifferentiation of the hair follicle consists of stages 6 to 8, in which hair follicles form the dermal papilla, root sheath, hair shaft, and eventually develop to maturity (3). These stages involve dynamic interactions of signaling pathways, including Wnt, Notch, and Sonic hedgehog, which coordinate cell proliferation, differentiation, and migration within the hair follicle (4). Diseases related to the process of hair follicle development, such as alopecia areata, hair loss, and gray hair, are hot topics of current research. But the treatment of these diseases still faces a lot of problems. For example, the current therapeutic drugs for hair loss, minoxidil and finasteride, require a longer period of use and have differences in individual validity (5). Traditional treatments for alopecia areata have limited efficacy and higher recurrence rate. New treatments such as JAKi and abatacept are associated with more side effects (6). For exploring more specific and effective treatments, there is an urgent need to find key targets that promote hair growth. Therefore, it is important to be clear about the mechanisms that regulate its growth, which triggers us to do this research.

In recent years, skin organoids have become a valuable tool for modeling skin structure and functions *in vitro*. Advances in stem cell biology have made it possible to generate skin organoids from pluripotent stem cells (PSCs), providing a powerful model for studying skin development. The skin organoids derived from human PSCs can be induced to generate hair follicles, offering a promising system for studying the cellular and molecular underpinnings of hair follicle development (7). The development of hair follicles in skin organoids is a multifactorial process, influenced by both intrinsic genetic factors and extrinsic environmental cues. Between days 55 and 75 of culture, skin organoids undergo the early formation of hair placode, hair matrix, and hair germ. During days 75 to 90 of differentiation, the hair placode and hair germ further differentiate into the hair peg. During days 110 to 130, the hair peg forms a more complex hair follicle containing sebaceous glands and root sheaths (8). Similar to *in vivo* hair follicle development, skin organoids require precise regulation of signaling pathways and cellular interactions.

We define and categorize the six developmental stages of hair follicles by corresponding to key morphological and molecular transitions observed during the hair follicle formation process. By D55, epithelial cell invagination forms a placode, and its interaction with mesenchymal cells initiates hair germ formation. By day 75, the hair germ deepens and forms a hair peg. This stage marks the transition from the initial hair germ into a more defined structure with columnar epithelial cells surrounded by a dermal papilla. By D90, the hair peg has developed further into a more recognizable hair follicle structure, with the root sheath and sebaceous glands starting to form and the epithelial and mesenchymal components of the hair follicle becoming more defined. By 140, the hair follicle exhibits initial maturation, characterized by the elongation of the hair shaft and the continued differentiation of the root sheath and dermal papilla. By 150, the hair follicle reaches full maturation. The hair shaft is fully formed, and the hair follicle is capable of producing hair in a more structured manner. The dermal papilla, root sheath, and sebaceous glands are fully developed, and the hair follicle has acquired the necessary components for mature, functional hair production. By D170, the hair follicle represents aging. At this point, the hair follicle begins to show signs of functional decline and evidence of cellular degradation in the follicle.

Spatial proteomics is an emerging field that enables the analysis of protein localization within tissues at a high resolution. Unlike traditional proteomics, spatial proteomics allows for the mapping of protein distribution in specific cellular compartments or regions of interest. This technique can reveal how protein localization changes during development and how proteins within a tissue interact in a spatially restricted manner (9). Recent studies have made it possible to map the spatial proteome of complex tissues like the skin, providing *in situ* analysis of specific functions of different structures (10). Spatial proteomics offers an exciting opportunity to enhance our understanding of hair follicle development within the skin organoid model. The application of spatial proteomics allows for the identification of key signaling proteins and structural components that regulate hair follicle development in a spatially resolved manner. By mapping spatial proteomics across different stages of hair follicle development, we can gain insights into how molecular signals are coordinated within specific regions of the hair follicle.

In this study, we apply spatial proteomics to map the general spatial proteome architecture profile of hair follicles at different developmental stages: D55 (hair germ formation), D75 (hair peg formation), D90 (hair follicle appearance), D140 (hair follicle initial maturation), D150 (hair follicle full maturation), and D170 (hair follicle aging). We identify specific and trending proteins as well as corresponding pathways to study the potential molecular mechanisms of hair follicle development. This study will contribute to our understanding of molecular events driving hair follicle formation and maturation at the proteomic level and may pave the way for new therapeutic strategies for hair loss and other skin disorders.

## Experimental Procedures

### Human-Induced PSC–Derived Organoid Culture

The human-induced PSC (hiPSC) line (nciPS02, RC01001-B, female; Nuwacell Biotechnologies Co, Ltd) was cultured on 6-well plates precoated with 1% Matrigel (BD). The PSCs were maintained in mTeSR1 (Stem Cell) medium with 100 μg/ml normocin (Invivogen) and also coated with 1% Matrigel (Corning). For differentiation, the hiPSC colonies were detached using Accutase Cell Dissociation Reagent (Gibco). The cells were collected in a single-cell suspension in Essential 8 Flex (Gibco) medium containing 20 × 10^−6^ M Y27632 (Selleck). The cells were seeded at a density of 3500 cells per 100 μl of medium per well in 96-well U-bottom plates (Corning). After 24 h of incubation at 37 °C, fresh E8 medium without Y27632 was added to bring the total volume to 200 μl per well, and the plates were incubated at 37 °C. Subsequently, the cell aggregates were cultured in an E6-based differentiation medium containing 2% Matrigel, 10 × 10^−6^ M SB431542 (Selleck), 4 ng/ml^−1^ basic fibroblast growth factor (R&D Systems), and 2.5 ng/ml^−1^ bone morphogenetic protein 4 (R&D Systems). On day 3, 200 ng/ml^−1^ LDN-193189 (bone morphogenetic protein inhibitor; Selleck) and 50 μg/ml^−1^ fibroblast growth factor were added to induce the formation of cranial neural crest cells. On day 12, all aggregates were transferred to 24-well low-attachment plates (Thermo Fisher Scientific) with 500 μl of organoid maturation medium, composed of a 1:1 mixture of F-12 (Dulbecco's modified Eagle's medium/F-12; Gibco) and neurobasal medium (Gibco), 1× GlutaMax (Gibco), 0.5 × B-27 minus vitamin A (Gibco), 0.5× N-2 (Gibco) supplements, 0.1 × 10^−3^ M 2-mercaptoethanol (Gibco), and 100 μg/ml^−1^ normocin, containing 1% Matrigel, to induce epidermal self-assembly. Half of the medium was replaced on day 16 after differentiation (11). Skin organoids at culture durations of D55, D75, D90, D140, D150, and D170 were collected.

### Laser Capture Microdissection of Hair Follicles of Skin Organoid Samples

Laser capture microdissection was performed using a laser microdissection system from Molecular Machines and Industries (MMI CellCut Laser Microdissection; MMI GmbH), which was controlled by the MMI Cell Tools software from the same company. The hair follicles of skin organoid samples of six developmental stages were obtained according to the following procedure. First, skin organoids were embedded in paraffin. Paraffin sections of skin organoids were cut and mounted on MMI MembraneSlides (MMI GmbH). After calibrating the laser focus and power, hair follicles of six developmental stages were cut successively. Finally, hair follicle samples of six developmental stages were obtained and attached to the EP tube cover (10, 12).

Samples were lysed by adding 10 μl of lysis buffer containing 1% sodium deoxycholate, 10 mM Tris(2-carboxyethyl)phosphine, 40 mM 2-chloroacetamide, and 100 mM triethylamine bicarbonate (pH 8.0), followed by thorough mixing. Reduction and alkylation were performed by incubating the samples at 95 °C for 10 min, then cooling them to room temperature. Samples were subsequently sonicated for 20 min with sealing film to ensure consistent disruption. Proteins were digested by adding trypsin and incubating at 37 °C for more than 4 h. Peptides were desalted using a C18 column, which was activated with 100% acetonitrile and equilibrated with 0.1% formic acid. After sample loading, impurities were removed by washing with 0.1% formic acid, and peptides were eluted with 70% acetonitrile. The eluate was collected and lyophilized for subsequent analysis.

### Mass Spectrometry Analysis

The mass spectrometry (MS) analysis was conducted with a timsTOF Pro2 mass spectrometer (Bruker Daltonics) equipped with a Captive Spray nanoelectrospray ion source and coupled to a nanoLC system. The purified peptides were redissolved in 10 μl of mobile phase A (100% H_2_O with 0.1% formic acid) and centrifuged at 14,000*g* for 20 min at 4 °C. A total of 200 ng of the supernatant was injected for LC–MS/MS analysis. Chromatographic separation was performed using a C18 analytical column (Beijing Qinglian Biotech; 100 mm × 1.5 mm, catalog no.: QL-HPLC-100∗15) at a constant flow rate of 500 nl/min. The mobile phases consisted of buffer A (100% H_2_O with 0.1% formic acid) and buffer B (80% acetonitrile with 0.1% formic acid). The gradient elution program was as follows: 0 to 2 min, 5% to 10% B; 2 to 23 min, 10% to 24% B; 23 to 26 min, 24% to 36% B; 26 to 27 min, 36% to 95% B; and 27 to 30 min, 95% B. MS was performed in data-independent acquisition (DIA) mode. For DIA acquisition, the mass width was set to 10 Da. The mass range covered from *m/z* 368.5 to 1098.5 and was separated into 73 acquisition windows. No overlapping windows were acquired, and the total cycle time is 1.23 s. The resolution of the MS1 scan was set to 60,000 at *m/z* 1222. The accumulation time in the trapped ion mobility spectrometry tunnel was 50 ms, with an ion mobility range from 0.60 to 1.60 cm^2^/V·s. The capillary voltage was set to 1.5 kV. Raw MS data (.d format) were acquired for further analysis. To ensure the consistency and performance of the MS platform, human embryonic kidney 293T cell lysates (CRL-11268; Cell Resource Center, Peking Union Medical College) were used as standard samples and analyzed bidaily under identical conditions. A total of six human embryonic kidney 293T standard samples were analyzed, and pairwise Spearman's correlation coefficients were calculated across quality control samples. The average correlation coefficient was 0.98, indicating high reproducibility and stability of the MS system (10).

### Proteomic MS/MS Data Processing

The MS raw DIA data were processed using DIA-NN (version 1.9.0). Database selection was based on species relevance, annotation completeness, and sequence reliability. For this analysis, the *Homo_sapiens_SP_uniprot_20240307* database containing 20,434 protein entries was used. Search parameters were set as follows: trypsin as the proteolytic enzyme with a maximum of two missed cleavages was allowed; carbamidomethylation of cysteine was specified as a static modification; methionine oxidation and N-terminal acetylation were set as variable modifications. Peptide lengths were restricted to the range of 7 to 52 amino acids. Protein quantification was normalized using local regression models, and interference correction was applied at the MS2 level to exclude interfering fragment ions while retaining at least three fragments for quantification. The precursor mass tolerance was set to 20 ppm, and the fragment mass tolerance was set to 0.05 Da for MS2. False discovery rate control was performed using the mProphet method with a peptide-level false discovery rate threshold of 1% (10). The proteomics data generated in this study have been deposited to the ProteomeXchange Consortium *via* iProX (13, 14) repositories with identifier PXD062716 (shared URL: https://www.iprox.cn/page/PSV023.html;url=1744101263014ixH1, password: A6yX) and Supplementary Table 1.

### Experimental Design and Statistical Rationale

Each experimental group consisted of three skin organoids of the same batch of cultures, as the samples collected from a single organoid cannot be gathered in sufficient quantities. In addition, within each group, we performed three technical replicates (as detailed in Supplementary Table 1) to account for potential technical variation and to minimize bias introduced by the MS platform. All data preprocessing, statistical analyses, and the majority of visualizations were conducted using R (version 4.4.1, R Foundation for Statistical Computing). Protein quantification was performed following normalization to the total intensity, scaled by a factor of 1,000,000, and subsequently log2-transformed. Principal coordinates analysis was applied to proteins with valid values across all samples, utilizing the ggbiplot package (version 0.55) (15). To assess statistical differences in protein intensities across six time points, a one-way ANOVA was conducted, with significance defined as an adjusted *p* value <0.05 (Benjamini–Hochberg correction) and a fold change exceeding 1.5. For a protein to be classified as an increasing-trend protein, its mean expression had to exhibit a stepwise increase across three consecutive groups, with each step showing a fold change greater than 1.5 on a log2 scale. Conversely, for a protein to be classified as a decreasing-trend protein, its mean expression had to exhibit a stepwise decrease across the same groups, with each step showing a fold change greater than 1.5 on a log2 scale. Heatmaps of significant proteins were generated using ggplot2 (version 3.5.1) (16) and ComplexHeatmap (version 2.20.0), employing Pearson’s distance metric and complete-linkage clustering (17). Functional enrichment analyses were carried out with the clusterProfiler package (version 4.12.6) (18), classifying proteins according to Gene Ontology terms, including biological processes, cellular components, and molecular functions. Protein interaction networks were constructed in Cytoscape (version 3.10.3, Cytoscape Consortium) (19), incorporating interaction data retrieved from the STRING database (20). To characterize extracellular matrix (ECM) components, proteins were annotated using the Matrisome database (http://matrisomeproject.mit.edu/proteins/) (21), which categorizes core ECM proteins (*e*.*g*., collagens, proteoglycans, ECM glycoproteins) and ECM-associated proteins (*e*.*g*., ECM regulators, ECM-affiliated proteins, secreted factors). In addition, the circlize package (version 0.4.16) was employed to generate circular visualizations of proteins exhibiting differential expression across multiple biological processes over six time points (22).

## Results

### General Spatial Proteome Profile of Hair Follicle of hiPSC-Derived Skin Organoid at Different Developmental Stages

**Fig. 1:**
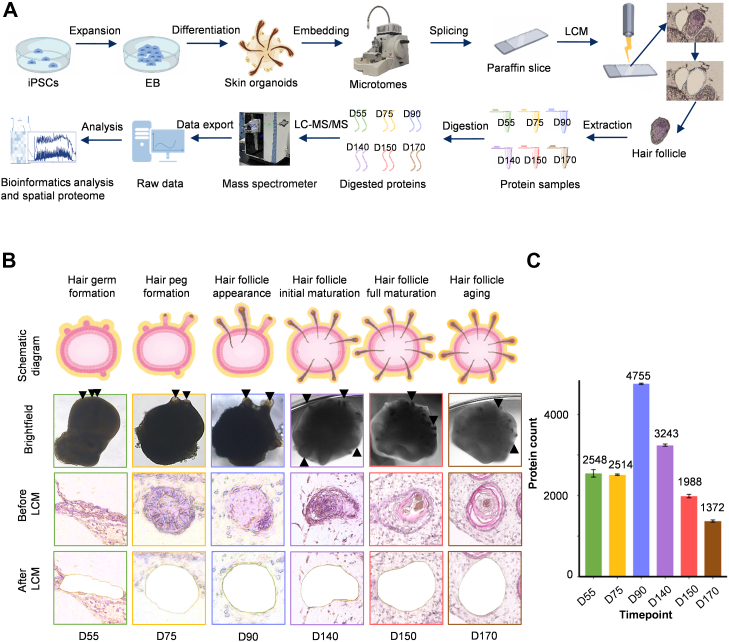
**Workflow of the experiment and demonstration of hair follicles at different developmental stages**. *A,* schematic diagram of the experimental workflow of the skin organoid culture, paraffin slice making, laser capture microdissection (LCM), spatial proteomics, and bioinformatics of hair follicles of six developmental stages, including D55 (*green*), D75 (*yellow*), D90 (*blue*), D140 (*purple*), D150 (*red*), and D170 (*brown*). Expansion from human-induced pluripotent stem cells (hiPSCs) forms embryoid bodies (EBs), which differentiate to generate skin organoids. Hair follicle samples were obtained from skin organoids by embedding, paraffin sectioning, and LCM. The dissected samples from each stage were extracted, digested, and analyzed using LC–MS/MS. *B*, the first row is a schematic diagram of hair follicles at different developmental stages, from *left* to *right* in order of hair germ formation (D55), hair peg formation (D75), hair follicle appearance (D90), hair follicle initial maturation (D140), hair follicle full maturation (D150), and hair follicle aging (D170). The second row is a bright field diagram of hair follicles at different developmental stages, from *left* to *right* in order of hair germ formation (D55), hair peg formation (D75), hair follicle appearance (D90), hair follicle initial maturation (D140), hair follicle full maturation (D150), and hair follicle aging (D170). *Black solid triangles* point to hair germ, hair peg, and hair follicle structures in the bright field diagram. The third row is a diagram of hair follicles before LCM at different developmental stages, from *left* to *right* in order of hair germ formation (D55), hair peg formation (D75), hair follicle appearance (D90), hair follicle initial maturation (D140), hair follicle full maturation (D150), and hair follicle aging (D170). The fourth row is a diagram of hair follicles after LCM at different developmental stages, from *left* to *right* in order of hair germ formation (D55), hair peg formation (D75), hair follicle appearance (D90), hair follicle initial maturation (D140), hair follicle full maturation (D150), and hair follicle aging (D170). Different colored boxes represent different developmental stages; *green* represents hair germ formation (D55), *yellow* represents hair peg formation (D75), *blue* represents hair follicle appearance (D90), *purple* represents hair follicle initial maturation (D140), *red* represents hair follicle full maturation (D150), and *brown* represents hair follicle aging (D170). *C*, number of proteins identified in D55, D75, D90, D140, D150, and D170 of hair follicle during skin organoid development.

In order to investigate the molecular mechanisms of human hair follicle development, we establish a skin organoid model to map the proteome profile of specific hair follicle structures. The skin organoids were constructed as in our previous study by using hiPSCs to form embryoid bodies and thus differentiate into skin organoids at different developmental stages (10). Microdissected samples of skin organoids were produced by the frozen section technique (Fig. 1*A*). Subsequently, samples of hair follicles at different developmental stages, including hair germ (D55), hair peg (D75), hair follicle appearance (D90), hair follicle initial maturation (D140), hair follicle full maturation (150), and hair follicle aging (D170), were obtained by laser capture microdissection to conduct an insightful study of hair follicle development (Fig. 1, *A*–*B*). Using spatial MS, we obtained raw data on digested hair follicle proteins for further data analysis. We analyzed the protein abundance in each developmental stage, which was combined with precise anatomical location information to construct a spatially resolved proteome map of hair follicle development (Fig. 1*A*). As a result, 2548 proteins in D55, 2514 proteins in D75, 4755 proteins in D90, 3243 proteins in D140, 1988 proteins in D150, and 1372 proteins in D170 in total were identified (Fig. 1*C*). The stage of hair follicle appearance (D90) had the highest amounts of proteins and subsequently gradually declined with development. At D55 and D75, the hair follicle was in an early stage of development, with the formation of early hair germs and hair pegs, and protein synthesis had not reached its peak (Fig. 1*C*). At D90, the hair follicle began to appear, so protein synthesis may be more active (Fig. 1*C*). More and more hair follicles tended to form and mature. Subsequently, the metabolic activity of the hair follicle decreases, and the hair follicle growth slows down, which may lead to a decline in protein synthesis (8). There were 1358 proteins that appeared in hair follicle samples from all six stages (supplementary Fig. S1*A*), and these highly conserved shared proteins may be essential for hair follicle development and involved in core biological processes. There were 903 unique proteins in D90, showing a characteristic protein expression pattern during this stage (Supplementary Fig. S1*A*). The depth of protein hitting spectra was presented by measuring protein abundance, providing a visual comparison of protein expression levels at different hair follicle developmental stages. The curves at different stages were similar in overall shape, with a gradual decrease in protein intensity as protein rank increases, but some differences remain. At the same protein intensity levels, D90 exhibited the highest number of low-abundance proteins and D170 exhibited the lowest number of low-abundance proteins, with the sequence of counts aligning with the total protein counts (Supplementary Fig. S1*B*). The total amounts of D90 and D140 proteins were higher, and the expression level of low-abundance proteins may have increased as a consequence.

**Fig. 2:**
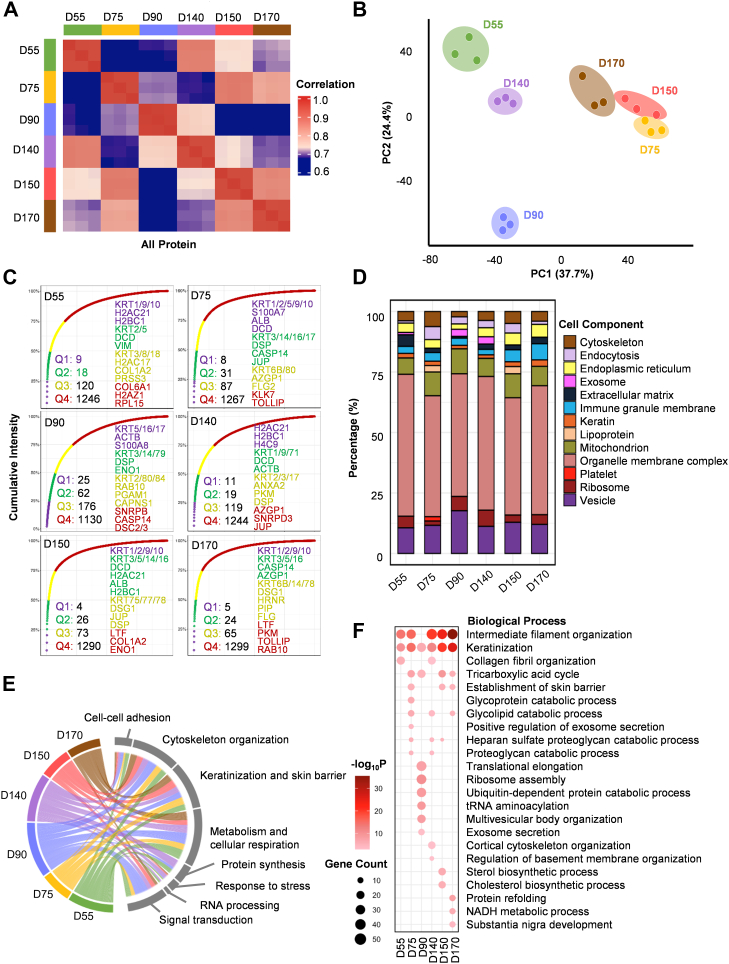
**General spatial proteome profile and functional characteristics of hair follicle of human-induced pluripotent stem cell–derived skin organoid at different developmental stages**. *A*, time-dependent analysis of the hair follicle proteomes based on the correlation matrix between the following stages of D55, D75, D90, D140, D150, and D170 of hair follicle during skin organoid development. Different colors represent different developmental stages; *green* represents hair germ formation (D55), *yellow* represents hair peg formation (D75), *blue* represents hair follicle appearance (D90), *purple* represents hair follicle initial maturation (D140), *red* represents hair follicle full maturation (D150), and *brown* represents hair follicle aging (D170). The *blue gradient* to *red boxes* represent low (0.5) to high (1.0) correlation. *B*, principal coordinate analysis of the proteome profile of each stage: D55, D75, D90, D140, D150, and D170 of hair follicle during skin organoid development. Different colors represent different developmental stages; *green* represents hair germ formation (D55), *yellow* represents hair peg formation (D75), *blue* represents hair follicle appearance (D90), *purple* represents hair follicle initial maturation (D140), *red* represents hair follicle full maturation (D150), and *brown* represents hair follicle aging (D170). *C*, cumulative protein abundances for each stage: D55, D75, D90, D140, D150, and D170 of hair follicle during skin organoid development and the total number of proteins constituting the quantiles (Q1: 25%, Q2: 50%, Q3: 75%, and Q4: 100%). *D*, cellular component analysis of proteins from each stage: D55, D75, D90, D140, D150, and D170 of hair follicle during skin organoid development. *E*, circos diagram shows proteins with top 10% expression abundance that are involved in multiple biological processes of hair follicles from each stage: D55, D75, D90, D140, D150, and D170 during skin organoid development. *Green*, *yellow*, *blue*, *purple*, *red*, and *brown lines* represent the correlations between multiple biological processes and each stage of D55, D75, D90, D140, D150, and D170 of hair follicle during skin organoid development, respectively. *F*, biological process enrichment analysis of all proteins in each stage: D55, D75, D90, D140, D150, and D170 of hair follicle during skin organoid development. *Circles* of different sizes represent the number of genes. *Gradient red boxes* indicate the –log_10_*p* value based on biological process enrichment.

After including all proteins for correlation analysis, correlation heat maps were obtained for six hair follicle developmental stages with 18 samples (Fig. 2*A*). Within the same stage, the samples showed a high positive correlation with very similar protein expression patterns (Fig. 2*A*). This indicated high confidence in the collection and selection of hair follicle samples. The protein expression patterns between D150 and D170 showed a relatively high correlation (Fig. 2*A*), indicating relatively similar protein expression patterns between hair follicle full maturation and aging. Meanwhile, the correlation between D90 and other developmental stages was comparatively minimal (Fig. 2*A*), suggesting that the protein expression pattern of hair follicle appearance exhibited notable divergence. From the principal coordinate analysis plot, the hair follicles in different developmental periods were more dispersed in distribution, indicating that there were differences in protein expression in hair follicles in different developmental stages. From hair germ to hair follicle maturation and aging, the proteome of hair follicles changed significantly, which may be related to changes in cell differentiation, proliferation, and function during hair follicle development (Fig. 2*B*).

Subsequently, proteins in the hair follicles in different developmental stages were divided into four parts according to the proportion of protein abundance (quantile Q1: 25%, Q2: 50%, Q3: 75%, and Q4: 100%) to identify the protein with specifically high abundance in different regions (Fig. 2*C*). Results showed that most of the highly expressed proteins (in Q1 or Q2) in different stages were keratins (KRT1, KRT9, and KRT10) (Fig. 2*C*), suggesting that keratinization is an essential procedure in the development of the hair follicle, which is consistent with previous studies (10, 23, 24). D150 and D170 had the most similar results (KRT1/2/9/10, KRT3/5/16) in high cumulative protein abundance (Q1 or Q2), indicating similar patterns of protein expression between hair follicle full maturation and aging (Fig. 2*C*). From the perspective of cellular components, we analyzed the cumulative percentage of cellular components of all proteins of hair follicles at each developmental stage (Fig. 2*D*). At each developmental stage, a relatively large number of proteins were expressed in mitochondria and vesicles (Fig. 2*D*). This suggested that cellular energy supply mediated by mitochondria and substance transport and secretory activities mediated by vesicles were more important for hair follicles at all stages (25, 26). D90 had the highest expression of mitochondria and vesicles (Fig. 2*D*). Hair follicles in D90 were just appearing and had a much higher energy demand and secretory activity. At D55, the organelle membrane complex was the largest percentage of cellular components (Fig. 2*D*). At D75 and D140, the percentage of lipoprotein was greater (Fig. 2*D*), indicating a greater need for lipid metabolism during hair peg formation and hair follicle initial maturation. At D90 and D140, the exosome cellular component was more predominant (Fig. 2*D*), suggesting that exosome-mediated substance transport and signaling were more prevalent during hair follicle initial maturation. From D90 to D170, the proportion of the immune granule membrane gradually increased (Fig. 2*D*), showing a progressive increase in immunoreactivity from hair follicle appearance to aging. Hair follicles were exposed to more external stimuli as they matured, thus, immune surveillance and local immune responses may gradually intensify. By D150 and D170, fully mature hair follicles began to age, and immune granule membranes may be associated with cell clearance, waste disposal, and tissue repair within the hair follicles.

Totally, these results indicate that a spatiotemporal proteomic map of hair follicles in skin organoids during the development process was first established. We found that energy demand and secretory activity are higher during hair follicle appearance, whereas lipid metabolic activity is higher during hair peg formation and hair follicle initial maturation.

### Functional Characteristics of Hair Follicle During Different Developmental Stages of Skin Organoid

To investigate the functional characteristics of hair follicles during development, we obtained a circos diagram reflecting biological functions with commonalities by enriching the pathways hosting the top 10% of expressed abundance proteins for each developmental stage (Fig. 2*E*). The results showed that proteins occupying the top 10% of abundance during hair follicle development belonged mostly to keratinization and skin barrier, metabolism and cellular respiration, and signal transduction pathways (Fig. 2*E*). Hair follicle development involves the interaction of multiple cell types and requires multiple signaling pathways to regulate cell proliferation, migration, differentiation, and death (4). Hair growth is a metabolically demanding process, and cellular respiration provides energy for hair follicle development and maturation (27). These results indicate that during hair follicle development, cells face stresses like reactive oxygen species generated metabolically that persistently exist (28). Meanwhile, the pathway of response to stress accounted for a relatively large proportion (Fig. 2*E*). The stress response pathway helps hair follicle cells cope with stresses such as oxidative stress.

In order to conduct in-depth research on the biological processes involved in hair follicle development, we conducted hierarchical clustering analysis and Gene Ontology analysis. Results showed that all proteins from hair follicles at varying developmental stages were obtained (Fig. 2*F*). Pathways of keratinization and intermediate filament organization were highly expressed in almost all stages of hair follicle development (Fig. 2*F*). Structures of the hair follicle express different types of keratins, some of which are regarded as markers of the hair follicle cycle (29). Catabolic pathways, like proteoglycan catabolic process, heparan sulfate proteoglycan catabolic process, glycolipid catabolic process, glycoprotein catabolic process, and tricarboxylic acid cycle, were expressed in D75, D140, and D150 (Fig. 2*F*). This suggested that these types of metabolism were more important during hair peg formation, hair follicle initial maturation, and hair follicle full maturation. Directly enriched proteins of D90 were primarily involved in the pathways of signaling and material exchange (exosomal secretion, multivesicular body organization) and protein synthesis (tRNA aminoacylation, ribosome assembly, and translational elongation) (Fig. 2*F*). Another pathway of the ubiquitin-dependent protein catabolic process *via* the multivesicular body sorting can remove unwanted or damaged proteins and maintain the balance of proteins in the cell to ensure normal cellular functions (30). RAB27A, STAM2, and AARS1 were significantly enriched and highly expressed in D90 hair follicles (Fig. 2*F*). Previous studies have highlighted the critical role of RAB27A in melanosome transport, with its expression observed during the anagen phase of the hair follicle cycle (31). *STAM2*, which is involved in signaling pathways, has been identified as a potential candidate gene for chemotherapy-induced alopecia (32). Our findings confirmed that STAM2 was highly expressed during hair follicle appearance, suggesting it may be involved in growth signaling mechanisms. In addition, mutations in *AARS1*, a gene crucial for protein synthesis, are associated with hair thiodystrophy (33), indicating that the hair follicle appearance may be linked to alterations in sulfur metabolism. Directly enriched proteins of D150 were primarily involved in the pathways of cholesterol biosynthetic process and sterol biosynthetic process (Fig. 2*F*). LSS and MSMO1 were significantly enriched and highly expressed in D150 hair follicles (Fig. 2*F*), where they were implicated in cholesterol synthesis. Previous studies have shown that LSS is localized to the endoplasmic reticulum, with mutations leading to sparse and fragile hair (34). MSMO1 deficiency has been linked to psoriasiform dermatitis (35). These results indicated that cholesterol synthesis at this stage plays a crucial role in the full maturation of hair follicles. Directly enriched proteins of D170 were primarily involved in the pathways of protein refolding (Fig. 2*F*). Protein folding–associated HSPA5 was highly expressed in D170 (Fig. 2*F*). Downregulation of HSPA5 was found to occur in alopecia areata, with diminished inhibition of apoptosis (36), indicating that reduced expression of this protein might hinder the proper refolding of proteins within the cell, exacerbating the stress response and leading to increased apoptosis and hair follicle dysfunction.

These results indicate that the most significant biological processes of intracellular protein enrichment during hair follicle formation and maturation are metabolism and cellular respiration and signal transduction pathways. Pathways of growth signaling, material exchange, and protein synthesis are highly enriched during hair follicle appearance.

### The Specific High Expression Protein Pattern Reveals Key Molecular Events of Hair Follicle Development

**Fig. 3:**
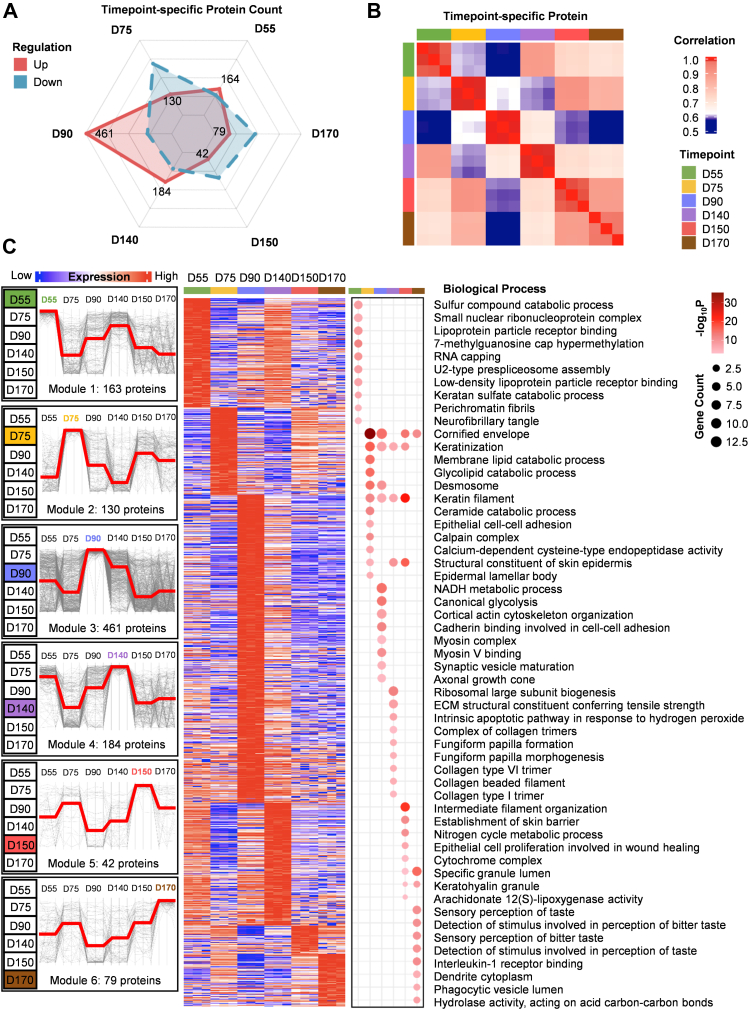
**The specific high expression protein pattern reveals key molecular events of hair follicle development**. *A*, number of proteins are specifically highly and lowly expressed in each stage: D55, D75, D90, D140, D150, and D170 of hair follicle during skin organoid development. *B*, time-dependent analysis of the specific high expression hair follicle proteomes based on the correlation matrix between the following stages of D55, D75, D90, D140, D150, and D170 during skin organoid development. Different colors represent different developmental stages; *green* represents hair germ formation (D55), *yellow* represents hair peg formation (D75), *blue* represents hair follicle appearance (D90), *purple* represents hair follicle initial maturation (D140), *red* represents hair follicle full maturation (D150), and *brown* represents hair follicle aging (D170). The *blue gradient* to *red boxes* represent low (0.6) to high (1.0) correlation. *C*, six protein modules reveal proteome specificity based on developmental stages using coexpression analysis. Each column within the developmental stages corresponds to a different hair follicle specimen. The *red and blue boxes* indicate the expression of proteins. *Left panel*, the coexpression patterns of the proteins in the six modules. *Right panel*, biological process enrichment analysis of all proteins in each stage: D55, D75, D90, D140, D150, and D170 of hair follicle during skin organoid development. *Circles* of different sizes represent the number of genes. *Gradient red boxes* indicate the –log_10_*p* value based on biological process enrichment. Different colors represent different developmental stages; *green* represents hair germ formation (D55), *yellow* represents hair peg formation (D75), *blue* represents hair follicle appearance (D90), *purple* represents hair follicle initial maturation (D140), *red* represents hair follicle full maturation (D150), and *brown* represents hair follicle aging (D170).

To identify the boundaries of the key features that delineate the different stages of hair follicle development and their specific biological significance, we performed hierarchical clustering analysis and Gene Ontology analysis to identify specific proteins of hair follicles at different developmental stages. These enriched specific proteins were linked to functional differences in the hair follicles at different developmental stages. The general number of specific proteins presented showed that D55 possessed 164 upregulated proteins, D75 possessed 130 upregulated proteins, D90 possessed 461 upregulated proteins, D140 possessed 184 upregulated proteins, D150 possessed 42 upregulated proteins, and D170 possessed 79 upregulated proteins (Fig. 3*A*). Hair follicles at D90 had the highest number of upregulated proteins, indicating more biological activity or change at this time point. According to our previous studies, D90 is a critical time point for the appearance of hair follicles in skin organoids, when various physiological functions of the hair follicle become active (11). The proliferation and differentiation of hair follicles and the initiation of hair growth may have led to the upregulation of a large number of specific proteins. The number of specific upregulated proteins in hair follicles of D55 and D75 was lower than that of D90 (Fig. 3*A*). This difference may be related to the fact that a complete hair follicle structure was not yet available at these stages. The hair follicles of D55 were predominantly hair germs, and the hair follicles of D75 were predominantly hair pegs (8, 11). Neither of them had reached their peak physiological function. Fewer specific upregulated proteins were observed in D150 and D170 (Fig. 3*A*), which correspond to the gradual maturation of hair follicle development and the slowdown of growth (37). We analyzed the correlation of specific highly expressed proteins at each stage to obtain a heatmap. The results indicated that D150 and D170 exhibited the highest correlation of specific highly expressed proteins, suggesting that hair follicle full maturation and aging share the most significant functional relevance (Fig. 3*B*). The specific highly expressed protein of D90 had the lowest correlation with other stages (Fig. 3*B*). It suggested that hair follicle appearance was a critical time point in hair follicle development with the highest specificity of functional expression.

To further explore the proteome specificity based on developmental stages, we generated six protein modules based on a heatmap of specific highly expressed proteins and compared their expression levels across the stages (Fig. 3*C*). The 163 enriched proteins in D55 (module 1) were mainly involved in RNA processing and splicing (perichromatin fibril, U2-type prespliceosome assembly, RNA capping, 7-methylguanosine cap hypermethylation, and small nuclear ribonucleoprotein complex) and sulfur catabolism (sulfur compound catabolic process and keratan sulfate catabolic process) (Fig. 3*C*), indicating that RNA processing and sulfur catabolism may be specifically highly expressed during hair germ formation. The 130 enriched proteins in D75 (module 2) were mainly involved in lipid metabolism (membrane lipid catabolic process, glycolipid catabolic process, and ceramide catabolic process) and cell–cell adhesion (desmosome and epithelial cell–cell adhesion) (Fig. 3*C*), indicating that lipid metabolism, especially ceramide metabolism, may have a specific role during hair peg formation. Module 3 of D90 represented the highest concentration of proteins (461 proteins), which were involved in various aspects of energy metabolism (NADH metabolic process and canonical glycolysis) and cytoskeletal dynamics (cortical actin cytoskeleton organization, myosin complex, and myosin V binding) (Fig. 3*C*). Hexokinase 1 (HK1) was D90-specific highly expressed proteins in the biological process of metabolism (Supplementary Fig. S2*A*), indicating that glycolysis mediated by HK1 may play a specific role during hair follicle appearance, which is consistent with the previous study that growing hair follicles have a high energy requirement, which is mainly realized through canonical glycolysis (27). Also, ENO1, PGK1, PGAM, PFKP, and PFKL, which were enriched in the pathway of metabolism, may also be possibly associated with hair follicle appearance (Supplementary Fig. S2*A*). The 184 enriched proteins in D140 (module 4) were involved in the intrinsic apoptotic signaling pathway responded to hydrogen peroxide, ECM structural constituents conferring tensile strength and collagen formation (collagen type VI trimer, collagen beaded filament, and collagen type I trimer) (Fig. 3*C*). The 42 enriched proteins in D150 (module 5) were involved in the establishment of the skin barrier, arachidonate 12(S)-lipoxygenase activity, and intermediate filament organization (Fig. 3*C*). We found that arachidonate 12-lipoxygenase (ALOX12B) in the biological process of metabolism is a D150-specific highly expressed protein (Supplementary Fig. S2*A*), indicating that ALOX12B may play a specific role during hair follicle full maturation.

**Fig. 4:**
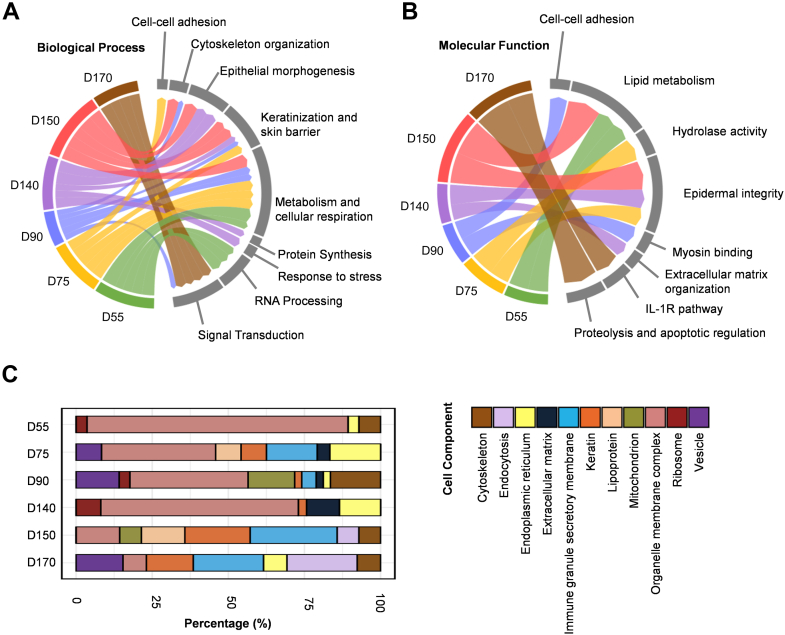
**The specific molecular events of hair follicle development based on the hair follicle developmental spatial proteome profile**. *A*, Circos diagram shows specific high expression proteins that are involved in multiple biological processes of hair follicles from each stage: D55, D75, D90, D140, D150, and D170 during skin organoid development. *Green*, *yellow*, *blue*, *purple*, *red*, and *brown lines* represent the correlations between multiple biological processes and each stage of D55, D75, D90, D140, D150, and D170 of hair follicle during skin organoid development, respectively. *B*, Circos diagram shows proteins that are involved in multiple molecular functions in hair follicles from each stage: D55, D75, D90, D140, D150, and D170 during skin organoid development. *Green*, *yellow*, *blue*, *purple*, *red*, and *brown lines* represent the correlations between multiple biological processes and each stage of D55, D75, D90, D140, D150, and D170 of hair follicle during skin organoid development, respectively. *C*, cellular component analysis of specific high expression proteins from each stage: D55, D75, D90, D140, D150, and D170 of hair follicle during skin organoid development.

We made circos diagrams of the enriched pathways from the perspective of biological process and molecular function for each stage of specific highly expressed proteins, reflecting the correlation of the different pathways with developmental stages and their weights (Fig. 4, *A*–*B*). From the perspective of cellular components, we analyzed the cumulative percentage of cellular components of specific proteins of hair follicles at each developmental stage (Fig. 4*C*). D55 was specifically associated with the RNA processing pathway (Fig. 4*A*). Hair follicles are capable of dynamically adapting to metabolic stress (38). D75 was specifically associated with the cell–cell adhesion pathway (Fig. 4*A*). Hair follicle development depends on the ability of keratinocytes to adhere to the basement membrane and migrate (39). The inability of keratinocytes to adhere properly within the hair follicle can result in a decrease in hair follicle density (40). As a result, for hair peg formation, normal expression of the cell–cell adhesion function may be required. D75 and D90 accounted for the largest proportion of the metabolic and cellular respiratory pathways, reflecting the specifically active energy metabolism during these stages (Fig. 4*A*). The stages associated with the cytoskeletal organization pathway are D90 (Fig. 4*A*). Significant alterations in hair follicle structure necessitate precise regulation of cytoskeletal dynamics (41). Hair follicle appearance is likely the stage during which changes in hair follicle morphology are most pronounced. In the circos diagram of molecular function, D90 specifically expressed the myosin binding function (Fig. 4*B*). D140 was specifically associated with the response to the stress pathway (Fig. 4*A*). Hair follicles are capable of dynamically adapting to metabolic stress (38). External stress induces the upregulation of interleukin (IL)-18 or IL-1β, resulting in the depletion of hair follicle's stem cells (42). Our findings indicated that initial maturation of hair follicles exhibits an active stress response, which may be linked to the heightened metabolic activity of the hair follicle growth. D140 specifically expressed the ECM organization function (Fig. 4*B*). The ECM, as part of the niche, is important in regulating hair follicle stem cell activity, particularly through epithelial–mesenchymal interactions (43, 44). Our results showed that hair follicle initial maturation was the stage at which the ECM played a major role. At D140, a large number of proteins performed functions in collagen (Fig. 4*C*). This indicates that at D140, many proteins related to collagen may play a significant role during the hair follicle initial maturation. D150 specifically expressed keratinization and skin barrier processes of biological process and epidermal integrity of molecular function (Fig. 4, *A*–*B*). In addition, keratin was the result of enriched cellular components to D150 (Fig. 4*C*). The molecular function of D170 was enriched for proteolysis and apoptotic regulation and the IL-1R pathway (Fig. 4*B*). Endocytosis and immune granule secretory membrane were the result of enriched cellular components to D170 (Fig. 4*C*). Studies have shown that the DNA damage response in hair follicle stem cells causes proteolysis of COL17A1, which triggers hair follicle aging (45). In the mouse model, the IL-1R signal can activate lipid droplets and NF-κB within hair follicle stem cells, further inhibiting Sonic hedgehog signal transduction, which ultimately accelerates the miniaturization of hair follicles and leads to hair loss (46). Therefore, our results demonstrate that the aging process of human hair follicles is associated with proteolysis and the IL-1R pathway and may be related to endocytosis and the immune granule secretory membrane.

Totally, these results indicate that the biological characteristics and processes involved in hair follicle development have temporal specificity. Among them, RNA processing and sulfur catabolism are specifically highly expressed for hair germ formation. Lipid metabolism is specific for hair peg formation. Proteolysis and the IL-1R pathway are specifically highly expressed in hair follicle aging.

### The Expression Patterns of Keratins and ECM Proteins During Hair Follicle Development in Skin Organoid

**Fig. 5:**
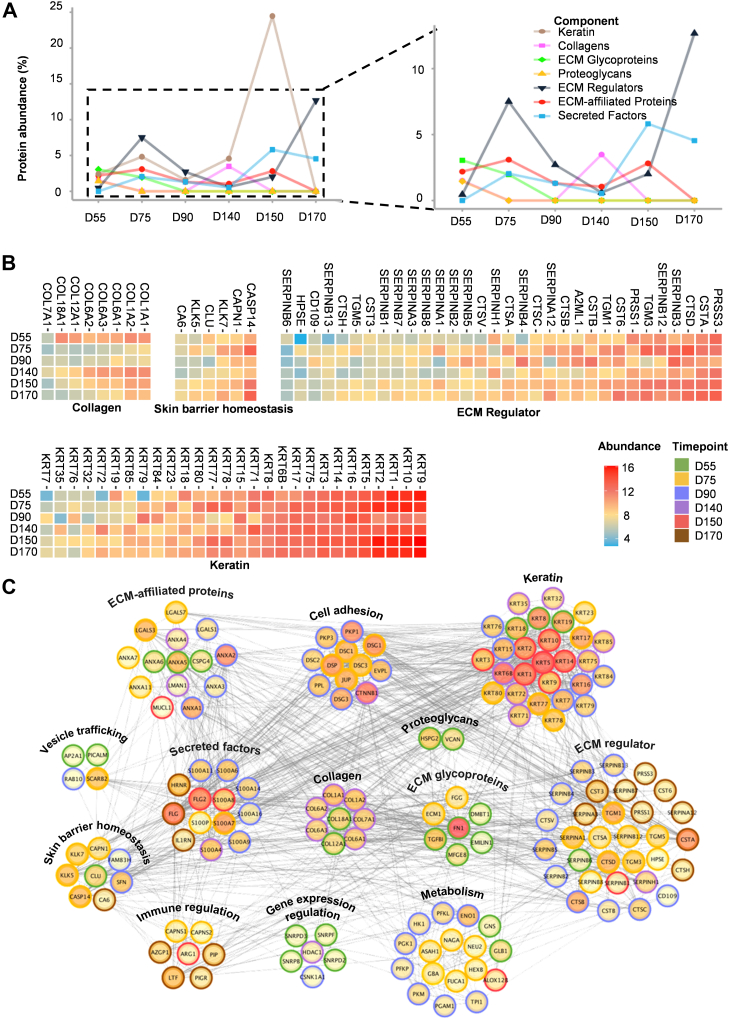
**Skin characteristic proteins and protein interaction analysis based on the hair follicle developmental spatial proteome profile**. *A*, protein abundance percentages of keratins, collagens, glycoproteins, proteoglycans, ECM regulators, ECM-affiliated proteins, and secreted proteins across six developmental stages: D55, D75, D90, D140, D150, and D170 of hair follicle during skin organoid development. The percentage was calculated by dividing the summed intensities of the proteins of interest by the summed intensities of all proteins identified in a specific stage. Source data are provided as a Source Data file. *B*, heatmap of specific high expression proteins from each stage: D55, D75, D90, D140, D150, and D170 of hair follicle during skin organoid development. The *columns* on the lower side of the heatmap indicate the different functional categories. The *top side* of the heatmap shows the names of the proteins. The *blue gradient* to *red boxes* represent low to high abundance. *C*, interaction network of proteins that are specifically highly expressed in hair follicles of each stage: D55, D75, D90, D140, D150, and D170 during skin organoid development. A map of the functional categories was used for primary biological process analyses. Different stages are labeled with different colors. *Green* represents hair germ formation (D55), *yellow* represents hair peg formation (D75), *blue* represents hair follicle appearance (D90), *purple* represents hair follicle initial maturation (D140), *red* represents hair follicle full maturation (D150), and *brown* represents hair follicle aging (D170). The depth of color in the *inner circle* represents the protein interaction strength with other proteins. *Gray solid lines* represent interactions between the proteins of each stage: D55, D75, D90, D140, D150, and D170 of the hair follicle during skin organoid development.

Due to the important role of ECM and keratins in hair follicle development (43), next, we determined the percentage change in the abundance of proteins related to ECM and keratins over time (Fig. 5*A*). In addition, we generated hierarchical presentation plots to depict the abundance of specific highly expressed proteins across different developmental stages (Fig. 5*B*).

The biomechanical components of keratinocytes, such as intermediate filament and keratin filament components, were mainly enriched in hair follicles. Keratins, which make up the cytoskeleton of epithelial cells, were highly expressed in the developmental stages of hair follicles, especially in D150 (Fig. 5*A*). A variety of keratins, such as KRT1, KRT2, KRT5, KRT9, KRT10, KRT14, KRT17, and KRT75, were highly expressed during the whole process of hair follicle development (Fig. 5*B*). KRT18 and KRT19 were highly expressed in D55 (Fig. 5*B*). KRT23, KRT71, KRT77, KRT78, and KRT80 were highly expressed in D75 and D150 (Fig. 5*B*). KRT7, KRT15, KRT76, KRT79, and KRT84 were highly expressed in D90 (Fig. 5*B*). KRT35 and KRT72 were highly expressed in D140 (Fig. 5*B*). These keratins are involved in maintaining cellular stability and the formation of the skin barrier.

PRSS3, CSTA, CTSD, and SERPINB3 were highly expressed during hair follicle development, associated with ECM regulators, which may be relatively important throughout the entire development process of hair follicles (Fig. 5*B*). RAB10 was consistently highly expressed during hair follicle development related to vesicle trafficking (Supplementary Fig. S2*A*). RAB10 marks and mediates the transport of intraluminal vesicles in the migrasome, which may be an important vesicle trafficking factor during hair follicle development (47). FN1, EMILIN1, and TGFBI were specifically highly expressed ECM glycoproteins, and ANXA6 was a specifically highly expressed ECM-affiliated protein in D55, which may play an important role in hair germ formation (Supplementary Fig. S2*B*). VCAN was a highly abundant proteoglycan, and HRNR was a highly abundant secreted factor of D55 (Supplementary Fig. S2*B*). CASP14 and KLK7 in the skin barrier homeostasis group were specifically highly expressed in D75 and D170 (Fig. 5*B*). Secreted factors like S100A7, S100A8, and S100A9 were highly expressed from D75 to D170 (Supplementary Fig. S2*B*). S100P was specifically a highly expressed secreted factor in D75 (Supplementary Fig. S2*B*). CSTB, SERPINB4, and SERPINB5 were specifically highly abundant proteins of D90 associated with ECM regulators (Fig. 5*B*). CSNK1A1 was highly expressed in D90 associated with gene expression regulation (Supplementary Fig. S2*A*). In the Angora rabbit model, CSNK1A1 was identified as a potential key regulatory factor for hair growth (48). Our results suggest that CSNK1A1 may also play a significant role in regulating gene expression during the key developmental process of human hair follicles. ANXA1 and PIGR were highly expressed in D90, associated with immune regulation (Supplementary Fig. S2*A*). PFKP and PFKL, which were enriched in the pathway of metabolism in D90, may also be possibly associated with hair follicle appearance (Supplementary Fig. S2*A*). ANXA1, ANXA3, and ANXA8L1 were specifically highly expressed ECM-affiliated proteins in D90, particularly ANXA8L1, which achieved a significant increase in protein abundance from D55 to D90 (Supplementary Fig. S2*B*). In addition, S100A14 was specifically a highly expressed secreted factor in D90 (Supplementary Fig. S2*B*). Collagens were highly expressed in D140 (Fig. 5*A*). COL1A1, COL1A2, COL6A1, COL6A3, and COL6A2 were highly abundant proteins of D55 and D140, which may be related to tensile strength changes (Fig. 5*B*) (49). The expression level of CSPG4 at D170 was significantly lower than that at several other hair follicle developmental stages, indicating that the effect of this ECM-affiliated protein on hair follicle aging may be relatively low (Supplementary Fig. S2*B*).

We made a protein–protein interaction network based on the specific highly expressed proteins at each stage, which was used to demonstrate the interaction relationship between proteins during hair follicle development (Fig. 5*C*). The network was categorized into various groups based on protein functions. The strong interaction between keratin and skin barrier homeostasis suggests that keratins primarily function in barrier maintenance, which is consistent with previous research (Fig. 5*C*) (50). This result demonstrates that the expression of KRT5, KRT14, and KRT15 in epidermal stem cells, along with KRT1 and KRT10 in mature keratinocytes, is closely linked to skin barrier function in developing hair follicles (Fig. 5*C*) (10). Our previous research indicated that KRT2, KRT76, KRT78, and KRT79 in the skin structure are associated with the epidermal barrier (10). The current result further confirms that the expression of these proteins in the hair follicle is also linked to skin barrier function (Fig. 5*C*). In addition, our study highlights the role of KRT6B, KRT8, KRT16, KRT17, and KRT19 in skin barrier homeostasis (Fig. 5*C*). According to the previous studies, mouse models with abnormal KRT16 expression exhibit skin lesions because of the excessive activation of damage-related molecules (51, 52). The acute disruption of the epidermal permeability barrier leads to a rapid increase in KRT17 expression in mouse models (53). Therefore, our results further support the potential role of KRT16 and KRT17 in maintaining the skin barrier in human hair follicle organoid models. Our results show that the interaction between the ECM regulator group and cell adhesion group is the most significant, indicating that this type of ECM protein may be more responsible for cell adhesion (Fig. 5*C*). This result indicates that the expressions of CSTA, CTSB, SERPINB3, SERPINB5, TGM1, TGM3, and TGM5 in the ECM regulator group are associated with the cell adhesion (Fig. 5*C*). CTSB is considered a candidate gene associated with keratolytic winter erythema, a condition characterized by periodic skin peeling (54). Our findings indicate that CTSB primarily participates in cell adhesion processes, a function that may play a significant role in the mechanism of skin desquamation (Fig. 5*C*). Previous studies have demonstrated the role of TGM1 as a biomarker for psoriasis and the involvement of TGM5 in the pathogenesis of peeling skin syndrome (55, 56). These findings highlight the crucial role of ECM regulators in the regulation of cell adhesion, which is essential for maintaining skin integrity and stability.

These results indicate the unique time-dependent functional support of keratins and ECM proteins in hair follicle development. The results highlight the importance of the barrier function of keratins during hair follicle development and the ECM–cell adhesion interaction in the occurrence of hair loss diseases.

### Dynamic Changes in a Time Series of Hair Follicle Development

**Fig. 6:**
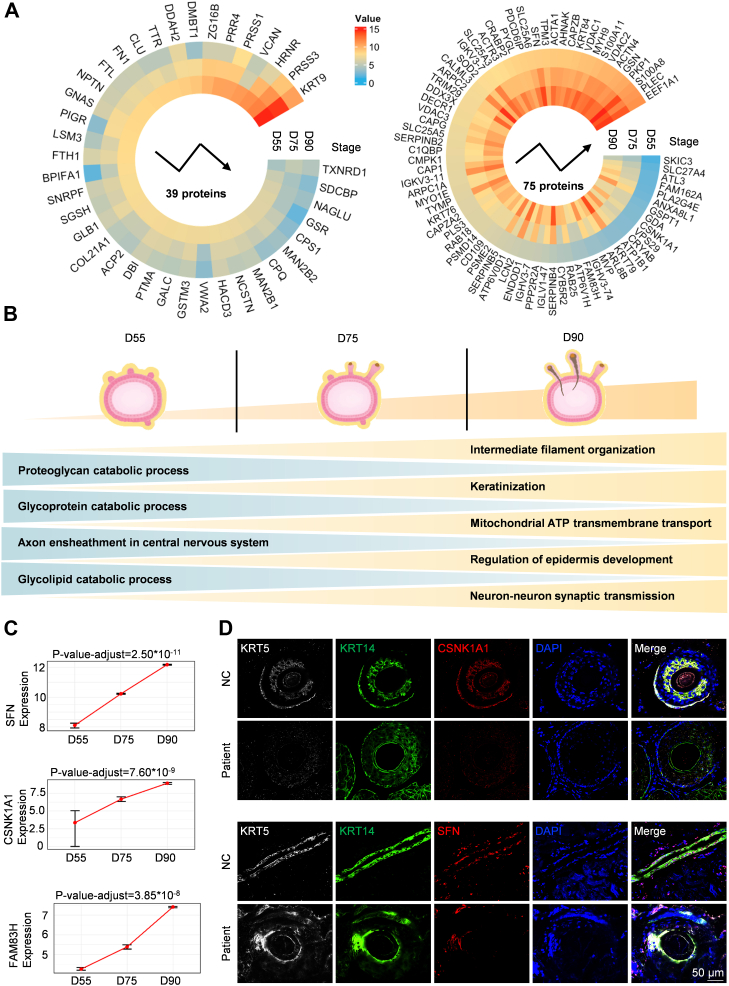
**Dynamic changes in a time series of hair follicle development**. *A*, upregulated and downregulated proteins in hair follicles at three stages: D75, D90, and D140. Each *circle* represents a stage. The *blue gradient* to *red boxes* represent low to high value of proteins of each stage: D55, D75, D90, D140, D150, and D170 of the hair follicle during skin organoid development. *B*, schematic representation of the hair follicle at three stages: D75, D90, and D140 during skin organoid development. *Yellow triangles* represent trend upward pathways of the hair follicle at three stages: D75, D90, and D140 during skin organoid development. *Blue triangles* represent trend downward pathways of hair follicle at three stages: D75, D90, and D140 during skin organoid development. *C*, expression of SFN, CSNK1A1, and FAM83H in hair follicles of three stages: D75, D90, and D140 during skin organoid development. *D*, immunofluorescence of KRT5, KRT14, SFN, CSNK1A1, and DAPI in the hair follicles of normal healthy people and patients suffering from cicatricial alopecia (the scale bar represents 50 μm).

In order to investigate alterations in biological processes that persist during hair follicle development, we selected D55, D75, and D90, the three most important stages of hair follicle formation, for trend analysis. Here, we focused on proteins that are trending upregulated or downregulated from D55 to D90 within the hair follicle. We identified 75 proteins upregulated with development and 39 proteins downregulated with development among these three stages (Fig. 6*A*). Our results indicated that the expression levels of intermediate filament organization, keratinization, mitochondrial ATP transmembrane transport, regulation of epidermis development, and neuron–neuron synaptic transmission–associated proteins increased gradually during hair follicle development (D55–D90) (Fig. 6*B*). Meanwhile, the expression levels of proteoglycan catabolic process, glycoprotein catabolic process, axon ensheathment in the central nervous system, glycolipid catabolic process–associated proteins decreased gradually (Fig. 6*B*), indicating that as hair follicle development progresses, the demand for these processes gradually decreases. Meanwhile, the gradual reduction of axonal wrapping in the central nervous system may reflect that neural development is in the early stage of hair follicle development. Our results are consistent with the previous research findings that basal sympathetic nerve activity is necessary for the activation of hair follicle stem cells and the entry of anagen. As the structure of hair follicles appears, the focus of development gradually shifts from the development of the nervous system to the keratinization process and hair shaft formation. As known, the hair germ gradually forms the hair peg and then the hair follicle (11). From D55 to D90, results showed that the expression levels of keratin formed by the organization of intermediate filaments and keratinization process–associated proteins increase significantly (Fig. 6*B*), which is consistent with the notable feature of hair follicle formation. The development and formation of hair follicles is a highly active biological process involving a large number of cell divisions, proliferations, and tissue remodeling. Furthermore, we found that during the process from hair germ to hair follicle formation in hair follicles, the expression levels of the energy production demand of mitochondria-associated proteins gradually increase (Fig. 6*B*), resulting in a gradual increase in the expression of the mitochondrial ATP transmembrane transport pathway. The results indicated that to support these metabolic activities, cells need an adequate energy supply, and mitochondria need to adapt to higher metabolic demands. In addition, neurons play a significant role in the growth of hair follicles, cell proliferation, differentiation, and functional maintenance through synaptic transmission. Our results indicate that the development process of hair follicles from hair germ to appearance not only depends on local molecular signals but may also be regulated by the signal transmission of the nervous system.

As is known, activation of the Wnt–β-catenin signaling pathway is essential for fibroblast proliferation and hair follicle formation (57). Among the gradually upregulated proteins, SFN and CSNK1A1, which are important regulatory factors in the Wnt–β-catenin signaling pathway, primarily influencing Wnt signaling activity by regulating the stability of β-catenin (58, 59), were found to be gradually upregulated from D55 to D90 (Fig. 6*C*). A previous study has already demonstrated the role of SFN in the hair follicle bulge region. Heterozygous mice with SFN mutations exhibit abnormalities in the hair follicle stem cell cycle, resulting in severe defects in hair shaft differentiation and disruption of hair shaft morphogenesis (60). CSNK1A1, enriched in overlapping pathways of the Wnt signaling pathway between RNA-Seq and WGRS, has been identified as a potential key regulator of wool growth, but no human samples have been used (48). However, there is currently no direct evidence proving the role of SFN and CSNK1A1 in human hair follicle development. However, our results showed that the expression levels of CSNK1A1 and SFN were downregulated in cicatricial alopecia compared with the normal controls (Fig. 6*D*). Cicatricial alopecia is a type of hair loss disease characterized by permanent destruction and fibrosis of hair follicles because of perifollicular inflammation (61). Our results showed that CSNK1A1 and SFN exhibit abnormal expression in the hair follicles of patients with cicatricial alopecia, indicating the possibly important role of CSNK1A1 and SFN in the normal developmental process of hair follicles.

These results indicate the key biological processes of intermediate filament organization, mitochondrial ATP transmembrane transport, and neuron–neuron synaptic transmission from hair germ formation and hair peg formation, as well as their proteins involved in the formation and development of hair follicles. Especially SFN and CSNK1A1 may be essential for human hair follicle development.

## Discussion

It is difficult to get samples of human hair follicles from formation to maturity for analysis. There are also limitations to the current mouse model, as the hair follicles formed differ from those of human origin. The growth cycle of mouse hair follicles is much shorter than that of humans. In the culture system of mouse skin organoids, complete hair follicle structures were formed at D20–D30 (62). In addition, the regenerative capacity of mouse hair follicles is also stronger. Human-derived cells showed considerably lower hair-inducing ability compared with mouse-derived cells (62). Here, based on the skin organoids from human PSCs, we first utilized spatial proteomics to investigate the developmental law and the key proteins’ characteristics during hair follicle development in skin organoids at different culture stages: D55, D75, D90, D140, D150, and D170.

Our study identified several stage-specific proteins that exhibited high expression at particular developmental stages. At D55, proteins associated with early hair follicle formation, such as those involved in RNA processing and sulfur catabolism, were significantly enriched. These proteins are essential for the initial organization of hair follicles and the establishment of the hair germ. MiRNA is a product of RNA processing that plays a regulatory role in the normal function of hair follicle bulge stem cells, particularly in the induction of hair follicle formation (63). RNA methyltransferase, present in hair follicle stem cells, is required for balanced stem cell self-renewal, and depletion of it results in quiescent and aberrant stem cells (64). The addition of methionine had been shown to significantly increase the expression of genes, such as Wnt10b, β-catenin, Sonic hedgehog, HGF, EGF, and Noggin. Methionine could promote hair follicle development by Wnt–β-catenin signaling (65, 66). Meanwhile, cysteine proteinase is essential for regular hair follicle morphogenesis and cycling (67). The specific high expression protein GLB1 is involved in glycosaminoglycan metabolism and is associated with different hair diameters (68). Our study further demonstrated the role of RNA processing and sulfur catabolism for hair follicle stem cells during the hair germ formation. As hair follicles transitioned to D75, a notable increase in proteins linked to lipid metabolism, including those involved in glycolipid catabolism and ceramide catabolism, was observed. Maintenance of cholesterol homeostasis is associated with hair follicle cell proliferation and differentiation (69). Meanwhile, abnormal ceramide metabolism can lead to abnormal development and even progressive loss of hair follicle stem cells (70, 71). This suggests that lipid metabolism plays a pivotal role during early hair follicle development of hair peg formation. At D90, when the hair follicle forms, proteins related to energy metabolism and cytoskeletal dynamics, such as HK1, PFKL, and ENO1, were highly expressed. Downregulation of HK1 is associated with chronic restraint stress-induced hair growth inhibition (72). *PFKL*, associated with normal hair follicle development and cycle, is a genetic candidate for aplasia cutis congenita (73). ENO1 is an important enzyme in UV-induced skin photoaging (74). This finding is consistent with the high metabolic demand required for hair follicle growth during hair follicle appearance. As the hair follicle matures, especially at D140, proteins associated with collagen formation and ECM proteins conferring tensile strength, such as COL1A1 and COL6A1, were notably expressed. Previous studies have focused on changes in tensile strength during wound healing and scar formation (49). This suggests that the collagen fiber formation may correlate with altered ECM tensile strength and is important for hair follicle initial maturation. By D150 and D170, proteins involved in skin barrier formation, arachidonate 12(S)-lipoxygenase activity, and intermediate filament organization were elevated, with ALOX12B highly expressed. Mutations in *ALOX12B* resulted in impaired keratinization, thus causing autosomal recessive congenital ichthyosis (75). Type I keratin intermediate filaments are specifically expressed in the IRS. They are essential for the formation of hardened structures within the IRS that provide support and protection for the hair shaft (76). D150 has formed a fully mature hair follicle structure, and the hair shaft continues to lengthen (3), which may account for the specific high expression of intermediate filament organization.

Based on the aforementioned results, we found that D90 possesses a large amount of protein compared with other developmental stages (Fig. 1*C*). By D90, the hair follicle began to appear from the hair peg, with the root sheath and sebaceous glands starting to form, indicating a phase of significant biological activity, as the hair peg transitions to a more complex structure. Studies have shown that during the formation of hair follicles by hair peg, the changes in the expression patterns of connexin 26 and connexin 43, as well as the increase in the number of gap junctions composed of connexin subunits, are closely related to the morphogenesis of hair follicles. Meanwhile, gap junctions are channels that allow for intercellular communication between adjacent cells, which are considered to play a key role in the regulation of cell proliferation and differentiation (77). This indicates that the D90 hair follicle has a higher requirement for intracellular communication, possibly to regulate protein synthesis, cell proliferation, and differentiation during hair follicle appearance. By D90, the cornified cell envelope formation begins to appear in the hair canal and the root sheath of the appearing hair follicle, and involucrin, loricrin, the precursor proteins, and transglutaminases are expressed actively, leading to the synthesis of keratins (78). Our results show that proteins involved in energy metabolism, cytoskeletal dynamics, and RNA splicing are notably elevated during D90, which correlates with the active formation of hair follicles. Therefore, the D90 stage is highly dynamic, with intense cellular proliferation, differentiation, and protein synthesis requirements, requiring substantial metabolic energy and signaling coordination to support the formation of essential structural components like keratins and ECM proteins. This reinforces the interpretation of the high protein expression at this stage as an essential part of hair follicle development. This observation supports the understanding that during hair follicle appearance, hair follicles are in a state of high cellular proliferation and differentiation.

Interestingly, we have discovered more patterns and potential roles of ECM protein expression during hair follicle development. Results reveal that ECM proteins, such as TGFBI, VCAN, HRNR, and FN1, play specifically significant roles in D55 in activating the necessary signaling pathways and promoting hair germ formation (Supplementary Fig. S2*B*). TGFBI has been proven to enhance the growth and function of epidermal stem cells by activating the Wnt pathway, making it an important basement membrane ECM protein (10). Therefore, for the D55 hair follicle, the basement membrane ECM plays a regulatory role in the growth of epidermal stem cells, promoting the growth of the hair germ. *VCAN* is a key target gene in the Wnt–β-catenin signaling pathway, and its upregulation has been shown to promote hair growth (79, 80). Our findings suggest that VCAN may facilitate hair germ formation during the early stages of hair follicle development, which may provide valuable insights for the development of treatments for hair loss. Studies have shown that HRNR is highly expressed in the skin and hair follicles of healthy humans, but its expression is reduced in the lesions of patients with psoriasis and atopic dermatitis (81). Our findings suggest that HRNR may primarily play a role in the formation of hair germ and influence the early stages of hair follicle development. In addition, our study suggests that ECM proteins, such as PRSS3, S100A7, and S100A8, play a regulatory role throughout almost the entire development process of hair follicles (Fig. 5*B*, Supplementary Fig. S2*B*). The mesotrypsin encoded by the PRSS3 gene can promote the shedding of keratinocytes in the skin, playing a crucial role in skin desquamation and barrier function maintenance (82). We found that it may also affect the formation and distribution of keratinocytes during the development of hair follicles. S100A7 has been shown to be involved in the antimicrobial defense system present in the hair follicle epithelium of normal human scalps (83). Our findings suggest that human hair follicles may begin to develop antimicrobial defense capabilities at the hair peg formation stage, and this function is maintained throughout development. S100A8 plays a role in promoting hair follicle stem cell proliferation by mediating the Wnt–β-catenin signaling pathway against androgenic alopecia (84). The continuous expression of S100A8 from D75 to D170 in human hair follicle development models suggests that S100A8 may serve as a potential target protein for the treatment of androgenic alopecia.

Our study offers insights into the molecular mechanisms of hair follicle development. These insights could be crucial in understanding hair growth cycles and the biological processes that go awry in hair-related disorders like alopecia. STAM2 was significantly enriched and highly expressed in D90 hair follicles, suggesting its involvement in hair follicle appearance (Fig. 2*F*). STAM2 has been identified as a potential candidate gene for chemotherapy-induced alopecia (32), indicating that the pathogenesis of chemotherapy-induced alopecia may be focused on the stage of hair follicle appearance. By understanding the function of STAM2 during the hair follicle formation stage, therapeutic strategies targeting this stage may be developed in the future to alleviate or prevent chemotherapy-induced hair loss. LSS was significantly enriched in D150 hair follicles, and its mutations caused sparse and fragile hair, suggesting its function in hair follicle maturation (Fig. 2*F*) (34). Therefore, the research on LSS provides new insights into the normal regulation of hair follicle maturation and hair growth. Therapeutic interventions targeting this protein may offer new treatment directions for sparse and fragile hair. The downregulation of HSPA5 indicated weakened inhibition of apoptosis, which was found to be related to alopecia areata (36). Our results show that HSPA5 may play a role in hair follicle aging and hair loss, and it may provide new ideas for the treatment of alopecia areata by targeting the expression or function of HSPA5 to regulate apoptosis (Fig. 2*F*). In addition, our findings confirm the crucial role of CSNK1A1 and SFN in normal hair follicle development and their association with cicatricial alopecia (Fig. 6, *C*–*D*). Although most forms of cicatricial alopecia can be definitively diagnosed based on clinical presentation during the acute phase of the disease, diagnosis may prove challenging during the subacute, early, or late stages of the disease (61). Downregulation of SFN and CSNK1A1 may serve as potential biomarkers for studying cicatricial alopecia and provide clues for understanding its molecular mechanisms. The insights obtained from this study have significant implications for hair follicle formation and maturation. Also, the results can contribute to the development of therapeutic strategies for hair loss and other hair growth disorders. By profiling the spatial proteome of hair follicle stages, we have identified critical proteins and pathways that regulate hair follicle growth, maturation, and immune response. These findings offer potential targets for developing regenerative therapies aimed at promoting hair regrowth and restoring hair follicle function. Furthermore, the detailed characterization of the spatial distribution of proteins provides a foundation for improving skin organoid models, which could be used for *in vitro* studies of hair follicle biology and for testing potential therapeutic interventions.

Our study still has some limitations, such as the selection of only a few developmental stages and the somewhat limited analytical techniques. In the future, more detailed stages should be added to better capture the dynamic changes in hair follicle development. In addition, integrating transcriptomics and epigenomics for multiomics analysis would provide a more comprehensive understanding of key biological pathways. Future studies could also focus on the interaction between these proteins and their potential as biomarkers or therapeutic targets for hair regeneration and skin disorders.

## Conclusion

Our study presents a detailed spatial proteomic analysis of hair follicle development across multiple stages in skin organoids: hair germ formation, hair peg formation, hair follicle appearance, hair follicle initial maturation, hair follicle full maturation, and hair follicle aging. We observe stage-specific protein expression patterns and dynamic changes of proteins in a time series of hair follicle development, highlighting key molecular processes involved in hair follicle formation and maturation. In addition, this study reveals the involvement of RNA processing, lipid metabolism, keratinization, and ECM organization at specific stages. The significant role of keratinization, intermediate filament organization, and neuron–neuron synaptic transmission during the hair follicle appearance from the hair germ is verified. Notably, this study indicates that CSNK1A1 and SFN exhibit abnormal expression in the hair follicles of patients with cicatricial alopecia, which further validates the role of CSNK1A1 and SFN in the normal developmental process of hair follicles. These findings contribute valuable insights into the biological mechanisms governing hair follicle development and provide a foundation for the development of therapeutic approaches aimed at hair regeneration and the treatment of hair-related disorders.

## Data Availability

The proteomics data generated in this study have been deposited to the ProteomeXchange Consortium *via* iProX (13, 14) repositories with identifier PXD062716 (shared URL: https://www.iprox.cn/page/PSV023.html;url=1744101263014ixH1, password: A6yX). The raw data described in this article are detailed in Supplementary Table 1.

## Supplemental Data

This article contains supplemental data.

## Conflictof Interest

The authors declare no competing interests.
