# Supplementary material for: Spatially Resolved Proteomic Mapping in Skin Organoid for Hair Follicle Development
Source: Mol Cell Proteomics. 2025 Dec 9;25(1):101482. doi: 10.1016/j.mcpro.2025.101482 (PMC12805098; doi:10.1016/j.mcpro.2025.101482)
Supplement: Supplementary Data [file mmc2.pdf]

Supplementary data

Spatially resolved proteomic mapping in skin organoid for hair follicle development

Luling Liang[1, *], MD, Jia Zhou[1, *], MD, Wenjuan Wang[2, *], Wenwen Wang[1], MD, Yi Liu[1], PhD, Jun Li[2], MD, Ling Leng[1, †], PhD

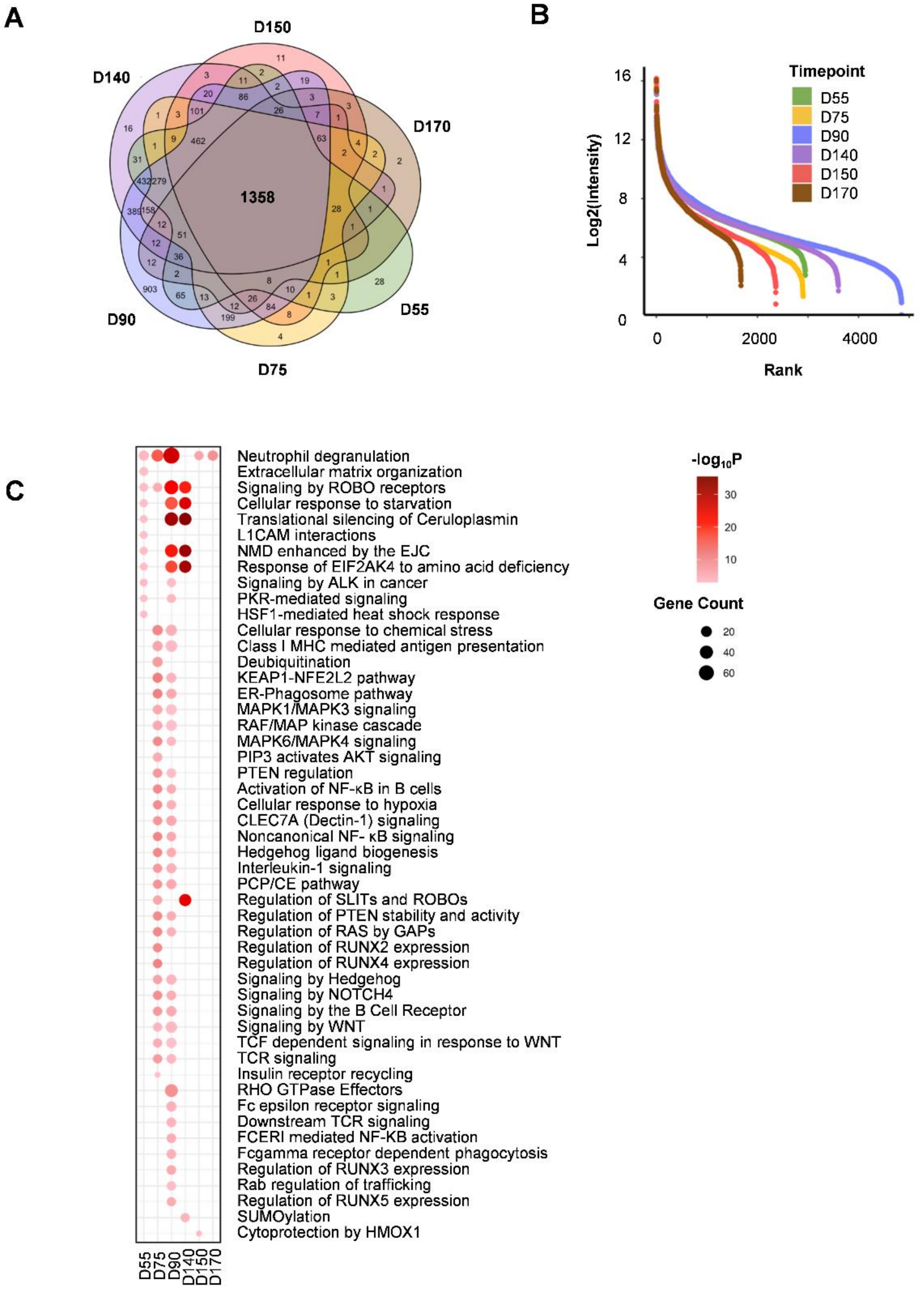

Supplementary Figure 1. Quantitative proteome profiling of spatially distinct protein signatures in hair follicle development.

Proteomic Mapping in Skin Organoid for Hair Development

(A) Venn diagrams show the overlap of the protein numbers identified in the hair follicles of each stage: D55, D75, D90, D140, D150, D170 of hair follicle during skin organoid development. Green represents hair germ formation (D55), yellow represents hair peg formation (D75), blue represents hair follicle appearance (D90), purple represents hair follicle initial maturation (D140), red represents hair follicle full maturation (D150), brown represents hair follicle aging (D170).

(B) Plots reflecting the depth of protein hitting spectra for each stage: D55, D75, D90, D140, D150, D170 of hair follicle during skin organoid development. Green represents hair germ formation (D55), yellow represents hair peg formation (D75), blue represents hair follicle appearance (D90), purple represents hair follicle initial maturation (D140), red represents hair follicle full maturation (D150), brown represents hair follicle aging (D170).

(C) Signaling pathways enrichment analysis of specific high expression proteins in each stage: D55, D75, D90, D140, D150, D170 of hair follicle during skin organoid development. Circles of different sizes represent the number of genes. Gradient red boxes indicate the $-\log_{10}$ p value based on biological process enrichment.

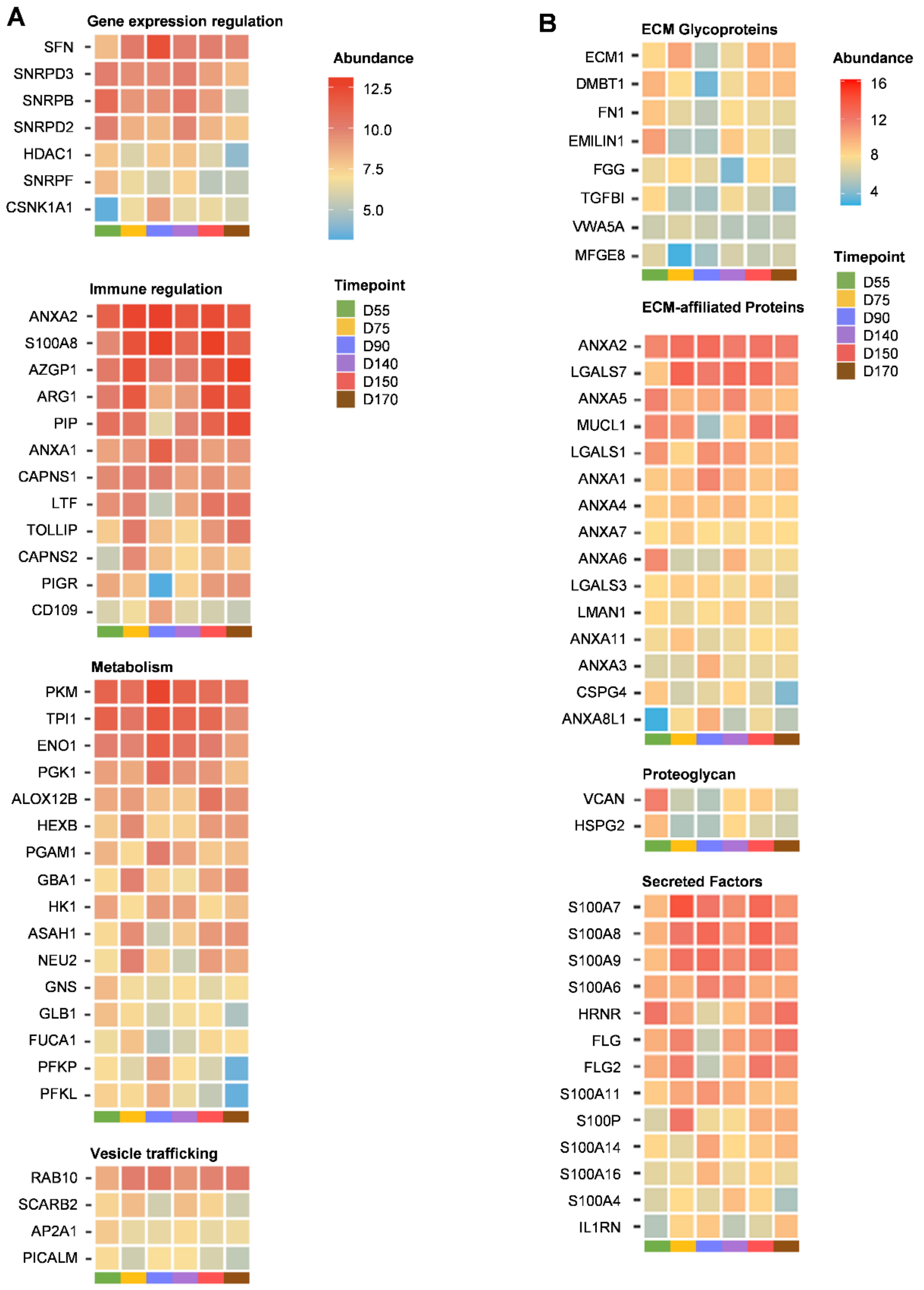

Supplementary Figure 2. Quantitative proteome profiling of specific highly expressed protein signatures in hair follicle development.

Proteomic Mapping in Skin Organoid for Hair Development

(A) Heatmap of specific high expression proteins from each stage: D55, D75, D90, D140, D150, D170 of hair follicle during skin organoid development. The columns on the upper side of the heatmap indicate the different functional categories (associated with the maintenance of basic cellular functions). The left side of the heatmap shows the names of the proteins. The blue gradient to red boxes represents low to high abundance.

(B) Heatmap of specific highly expressed proteins from each stage: D55, D75, D90, D140, D150, D170 of hair follicle during skin organoid development. The columns on the upper side of the heatmap indicate the different functional categories (associated with skin-specific functions). The left side of the heatmap shows the names of the proteins. The blue gradient to red boxes represents low to high abundance.
